# Identification and Developmental Expression of *Xenopus laevis* SUMO Proteases

**DOI:** 10.1371/journal.pone.0008462

**Published:** 2009-12-24

**Authors:** Yonggang Wang, Debaditya Mukhopadhyay, Smita Mathew, Takashi Hasebe, Rachel A. Heimeier, Yoshiaki Azuma, Nagamalleswari Kolli, Yun-Bo Shi, Keith D. Wilkinson, Mary Dasso

**Affiliations:** 1 Laboratory of Gene Regulation and Development, National Institute of Child Health and Human Development, Bethesda, Maryland, United States of America; 2 Department of Biochemistry, Emory University, Atlanta, Georgia, United States of America; Ludwig-Maximilians-Universität München, Germany

## Abstract

SUMO proteins are small ubiquitin-related modifiers. All SUMOs are synthesized as propeptides that are post-translationally cleaved prior to conjugation. After processing, SUMOs become covalently conjugated to cellular targets through a pathway that is similar to ubiquitination. Ubiquitin like protein proteases/Sentrin specific proteases (Ulp/SENPs) mediate both processing and deconjugation of SUMOs. The action of Ulp/SENPs makes SUMOylation a highly dynamic post-translational modification. To investigate how Ulp/SENPs are regulated in a developmental context, we isolated and characterized all Ulp/SENPs in *Xenopus laevis*. *Xenopus* possess homologues of mammalian SENP3, 5, 6 and 7. All of these enzymes reacted with HA-tagged vinyl sulfone derivatives of SUMO-2 (HA-SU2-VS) but not SUMO-1 (HA-SU1-VS), suggesting that they act primarily on SUMO-2 and -3. In contrast, *Xenopus* possess a single member of the SENP1/SENP2 subfamily of Ulp/SENPs, most closely related to mammalian SENP1. *Xenopus* SENP1 reacted with HA-SU1-VS and HA-SU2-VS, suggesting that it acts on all SUMO paralogues. We analyzed the mRNA and protein levels for each of the Ulp/SENPs through development; we found that they show distinct patterns of expression that may involve both transcriptional and post-transcriptional regulation. Finally, we have characterized the developmental function of the most abundant Ulp/SENP found within *Xenopus* eggs, SENP3. Depletion of SENP3 using morpholino antisense oligonucleotides (morpholinos) caused accumulation of high molecular weight SUMO-2/3 conjugated species, defects in developing embryos and changes in the expression of some genes regulated by the transforming growth factor beta (TGF-β) pathway. These findings collectively indicate that SUMO proteases are both highly regulated and essential for normal development.

## Introduction

SUMOs are among the most important and widely studied Ubiquitin-like proteins, with diverse roles in almost every critical aspect of nuclear function [1], [2], [3]. All SUMOs are synthesized as propeptides that are processed to reveal C-terminal diglycine motifs [4], [5]. Mature SUMO proteins become covalently conjugated to other proteins through isopeptide linkage between their C-termini and ε-amino groups of lysines within the targets. Budding and fission yeast both express a single SUMO protein, while vertebrate cells express three major SUMO paralogues (SUMO-1-3). After processing, mature SUMO-2 and -3 are ∼95% identical to each other, while SUMO-1 is ∼45% identical to SUMO-2 or -3. Where they are functionally indistinguishable, SUMO-2 and −3 will be collectively called SUMO-2/3 in this report. Modification of proteins by SUMOylation is transient and dynamic, due to the rapid turnover of conjugated species by SUMO proteases. Both processing and deSUMOylation are mediated through the action of the same family of proteases, called Ubl specific proteases (Ulp) in yeast [6] and Sentrin-specific proteases (SENP) in vertebrates [7]. Ulp/SENPs play a pivotal role in determining the spectrum of SUMOylated species, since they control both the final step in SUMO production and the half-life of conjugated species [8], [9].

Budding yeast has two Ulp/SENPs (Ulp1p and Ulp2p/Smt4p), and mammals have six (SENP1, 2, 3, 5, 6, and 7) [9]. Sequence alignment suggests that four mammalian SENPs (SENP1, 2, 3, 5) fall within a Ulp1p-related sub-family, while two mammalian SENPs (SENP6, 7) are more closely related to Ulp2p [9]. Ulp1p is encoded by an essential gene [6]; it is important for SUMO processing [6], 60S ribosomal particle export [10] and nuclear-cytoplasmic trafficking [11]. Human SENP1 and SENP2 are most closely related to each other, and can catalyze processing and deconjugation of all SUMO paralogues [12], [13], [14]. Human SENP3 and SENP5 are likewise most closely related to each other, but they show a strong preference for processing and deconjugation of SUMO-2/3 over SUMO-1 [15], [16], [17]. Ulp2p is not essential for vegetative growth, although it is important for sporulation [18]. Ulp2p appears to act in deconjugation [19], especially for disassembly of poly-SUMO chains [20]. Human SENP6 and SENP7 show a strong preference for deconjugation of SUMO-2/3-containing species, particularly for substrates containing multiple SUMO-2/3 moieties, that may be analogous to the activity of Ulp2p in chain disassembly [21], [22].

The SUMO pathway is required for normal development of flies [23], fish [24], mice [25], worms [26], [27] and frogs [28], [29], [30]. SUMOylation has been particularly implicated in regulation of the transforming growth factor (TGF-β) pathway, a signal transduction cascade that is central to vertebrate development. The type I TGF-β receptor becomes SUMOylated in rodents in response to TGF-β, enhancing the receptor's interactions with the Smad proteins that transduce TGF-β pathway signals and allowing more efficient phosphorylation and activation of Smad2/3 [31]. Smad proteins themselves are also SUMO conjugation targets and interact with PIAS-family SUMO ligases [32], [33], [34], [35], [36], [37], [38]. In *Xenopus*, morpholino antisense oligonucleotides directed against the SUMO-1 mRNA disrupt axis formation in embryos and elongation animal caps treated with the TGF-β family member Activin [29]. The roles of individual SUMO pathway enzymes in controlling developmental events have not been well characterized.

To develop a better understanding of the developmental control of Ulp/SENPs, we have cloned a comprehensive set of Ulp/SENPs from *Xenopus laevis*. Interestingly, while clear homologues exist for mammalian SENP3, SENP5, SENP6 and SENP7, there was a single member of the SENP1/SENP2 branch of evolutionary tree in *Xenopus*, which we will call SENP1 here. SENP1 reacted with HA-tagged vinyl sulfone derivatives of both SUMO-2 (HA-SU2-VS) and SUMO-1 (HA-SU1-VS), suggesting that it recognizes all SUMO paralogues in a manner similar to mammalian SENP1. By contrast, all other *Xenopus* SENPs showed a strong preference for HA-SU2-VS, suggesting that they primarily act on SUMO-2/3. We analyzed the complement of SENPs present in eggs and early embryos, and found that SENP1, 3, 6 and 7 are detectably present in *Xenopus* eggs. SENP1 and 3 persisted throughout development, although there was some variation in SENP1 levels near the midblastula transition (MBT). SENP6 and 7 were also clearly detectable in early embryos but declined dramatically in abundance around Stage 26 (tail bud stage). Finally, we characterized the developmental function of the most abundant Ulp/SENP found within *Xenopus* eggs, SENP3. Depletion of SENP3 using morpholinos caused changes in the profile of SUMO-2/3 conjugated species, defects in developing embryos and changes in the expression of some genes regulated by the transforming growth factor beta (TGF-β) pathway. Our findings collectively indicate that SUMO proteases are both differentially regulated and essential for development.

## Results and Discussion

### Identification of *Xenopus* SENPs

As a prelude to developmental analysis of the Ulp/SENPs, we used *Xenopus laevis* and *Xenopus tropicalis* databases to identify cDNAs and genomic sequences encoding Ulp/SENPs. We initially found five *Xenopus laevis* cDNAs encoding proteins with Ulp/SENP catalytic domains. Comparison of these sequences to mammalian SENPs indicated that they corresponded to SENP1, 3, 5, 6 and 7 (Table 1). These assignments were similar both in the context of the full sequences of each protein, and in the context of the catalytic domains alone, and were also consistent with earlier reports related to SENP proteins in *Xenopus* [17], [30]. The most remarkable feature of the *Xenopus laevis* sequences was the absence of an apparent homologue to mammalian SENP2. We considered the possibility that this cDNA was underrepresented within the database. We examined the genomic sequence of a closely related species, *Xenopus tropicalis*, and found that it had a similar complement of five Ulp/SENPs that lacked SENP2. Given the close evolutionary relationship of these two *Xenopus* species, it thus appears that the absence of a cDNA encoding *Xenopus laevis* SENP2 reflects the genuine absence of a corresponding gene.

**Table 1:** Characteristics of *X. laevis* SENPs.

*X. laevis*	*H. sapiens*	Homology Full length (%)	Homology Catalytic Domain (%)	SU1-VS Reactivity	SU-2-VS Reactivity	mRNA levels	Protein levels
SENP1 (AF526893)	SENP1 (BC045639)	53	80	+++	+++	Transient drop at MBT	Transient drop at MBT
	SENP2 (NM_021627)						
SENP3 (FJ416373)	SENP3 (NM_015670)	58	88	−	+++++	Present throughout development	Present throughout development
SENP5 (EU275408)	SENP5 (NM_152699)	39	91	−	++	Present throughout development	Below detection
SENP6 (FJ416371)	SENP6 (NM_015571)	51	61	−	++	Present throughout development	Decrease after stage 26
SENP7 (FJ416372)	SENP7 (BC129988)	37	54	−	+	Transient drop at MBT	Decrease after stage 26

SENPs sequences for *Xenopus laevis* (*X. laevis*) and *Homo sapiens* (*H. sapiens*) were from Genebank database (Accession number indicated below each protein). Homology analysis was performed using clustalw software from EMBL-EBI. The following domains were used for catalytic domain homology comparison: SENP1 (H.s. 427–644, X.l. 401–618); SENP3 (H.s. 386–574, X.l. 271–459); SENP5 (H.s. 567–755, X.l. 534–722); SENP6 (H.s. 637–1112, X.l. 641–1103); SENP7 (H.s. 662–1050, X.l. 501–901).

The reactivity of each SENP with HA-SU1-VS and HA-SU2-VS is indicated; “−” represents little or no adduct formation. “+” to “+++++” represents increasing percentages of the SENP converted to adduct in three independent experiments, with the latter representing total conversion within 30 minutes. mRNA and protein levels (Stage 1 to stage 43) are summarized from data in Figure 2 and Figure 3, respectively.

Mammalian SENPs can be organized into three sets that show pairwise sequence homology, as well as similar localization and biochemical properties [9]. Our findings suggest that this pairwise organization may be preserved in frogs, except in the case of the SENP1/2 sub-family of Ulp/SENPs. The absence of a SENP2 homologue in amphibians may suggest that the paired organization of these genes is a relatively recent feature of the evolution of vertebrate SUMO pathway, and that amphibians diverged from mammals before a second SENP1/2 sub-family member arose. Alternatively, the SENP2 homologue may have been lost after the divergence of amphibians and mammals. Two closely-related cDNAs encoding SENP1 have been reported in *Xenopus laevis*, designated xSENP1a and xSENP1b [30]. (The cDNA that we isolated corresponds to xSENP1a.) The existence of two cDNAs encoding SENP1 is likely reflect the pseudo-tetraploid status of the *Xenopus laevis* genome [39]. The proteins encoded by these cDNAs show a very high level of sequence similarity (88% within the catalytic domain; 86% overall) and both are much more closely related to human SENP1 than SENP2, making it improbable that either of them could represent a *Xenopus* homologue of SENP2. As discussed below, we observe primarily xSENP1a within early embryos. Given that there appears to be no true SENP2 in *Xenopus*, it is likely that all essential functions of mammalian SENP2 are performed by the remaining SENPs in frogs.

### Abundance and Paralogue Specificity of SENPs in *Xenopus laevis* Egg Extracts

**Figure 1:**
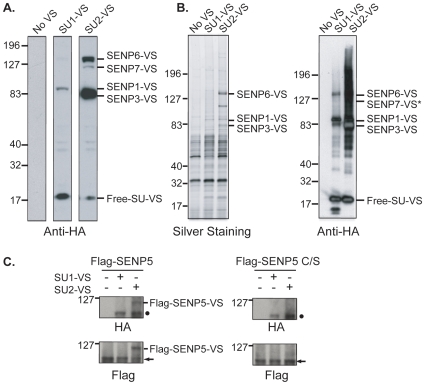
Abundance and paralogue preference of Ulp/SENP in XEEs. (**A**) XEEs were incubated with buffer (left), HA-SU1-VS (middle) or HA-SU2-VS (right), and subjected to SDS-PAGE and immunoblotting with anti-HA antibodies. Free SUMO vinyl sulfones and adducts with individual SENPs are indicated. (**B**) XEEs were incubated with buffer (left lanes), HA-SU1-VS (middle lanes) or HA-SU2-VS (right lanes). HA-tagged proteins were purified by affinity chromatography, resolved on SDS- PAGE and visualized by Silver staining (left panel) or by immunoblotting with anti-HA antibodies (right panel). Bands recognized by the anti-HA antibodies were identified by mass spectrometry as indicated to the right of each panel. “*” indicates that SENP7 was not identified by mass spectrometry, but could be readily detected by immunoblotting with an antibody specific to SENP7. (**C**) *In vitro* translated Flag-SENP5 and Flag-SENP5-C/S were incubated with HA-SU1-VS or HA-SU2-VS. The reactions were monitored by immunoblotting with anti-HA (Upper panel) or anti-Flag (Lower panel) antibodies. “▪” indicates the position of SENP1-VS in the upper panels. Arrow indicates the position of unreacted Flag-SENP5 or Flag-SENP5-C/S.

We analyzed the activity and abundance of SENPs, as well as their paralogue specificity within *Xenopus laevis* egg extracts (XEEs). This profile is useful not only because it reflects the contingent of SENPs present in the mature egg, but also because XEEs are valuable and well-established system that offers unique advantages for performing *in vitro* biochemical and cell biological studies [40]. To determine the spectrum of SENP activity within XEEs, we utilized vinyl sulfone derivatives of tagged SUMO-1 and -2 (HA-SU1-VS and HA-SU2-VS; [41]), which covalently and specifically react with the nucleophilic cysteine residue within the active sites of SUMO proteases. HA-SU1-VS or HA-SU2-VS was added to interphase XEEs. Vinyl sulfone reaction products were identified by Western blotting with antibodies against the HA tag (Figure 1A). The signal of individual bands on these Western blots reflects the relative abundance of the corresponding SENP proteins because HA-SU1-VS and HA-SU2-VS react with Ulp/SENPs in a manner that is both quantitative and irreversible.

The HA-SU1-VS reaction showed a single prominent band, with an apparent molecular weight of 90 kD (Figure 1A). The HA tag allowed affinity purification of this reaction product, which was subjected to SDS-PAGE. The region of the gel corresponding to this band was excised and digested with trypsin. The resulting peptides were subjected to mass spectrometry and the band was identified as SENP1 (Figure 1B; Figure S1). The peptides that we observed corresponded to the coding sequence of xSENP1a, which we will continue to call SENP1 throughout the remainder of this report. We did not find peptides unique to xSENP1b, and so were unable to validate its expression. However, the absence of such peptides does not exclude the possibility that xSENP1b may be present at some level.

Our finding suggests that XEEs have a single major SUMO-1-specific protease, SENP1, which falls into the SENP1/2 subfamily as the mammalian Ulp/SENPs with strongest activity against SUMO-1 [9]. Long exposures of the affinity purified material showed weak bands of mobilities that might be consistent with the formation of adducts between SENP6 or SENP3 and HA-SU1-VS. If so, the fact that these bands are much weaker than corresponding adducts in the HA-SU2-VS sample indicates inefficient recognition of SUMO-1 by these enzymes, consistent with the pattern observed in mammalian cells [22]. Incubation with HA-SU2-VS showed a much higher level of reactivity, with predominant band with an apparent molecular weight of 83 kD, and weaker bands with apparent molecular weighs of 123 and 144 kD (Figure 1A). Analysis of these samples after affinity purification of HA-tagged material showed that these bands corresponded to SENP3, SENP6 and SENP7, respectively. The sample also contained detectable amounts of SENP1 (Figure 1B). These findings are consistent with the strong SUMO-2/3 paralogue preference previously reported for mammalian SENP3, SENP6 and SENP7 [17], [21], [22].

SENP5 was the single SENP that we had found by sequence analysis but that we did not detect in XEEs as a HA-SU1-VS or HA-SU2-VS reaction product. We raised antibodies against SENP5 (Figure S2), and did not find detectable amounts of SENP5 by Western blotting of XEEs (data not shown), further suggesting that the levels of this protein are very low in unfertilized eggs. To determine the paralogue preference of *Xenopus* SENP5, we incubated an *in vitro* translated, Flag-tagged SENP5 with HA-SU1-VS and HA-SU2-VS (Figure 1C). Anti-Flag Westerns showed a decreased mobility of the Flag-SENP5 upon incubation with HA-SU2-VS. This band was also reactive with anti-HA antibodies, indicating that it corresponds to the Flag-SENP5 adduct with HA-SU2-VS. Notably, a mutant version of SENP5 in which an active site cysteine had been changed to serine did not show a similar form with reduced mobility, indicating that the appearance of this form required SENP5 to be catalytically active. We did not observe the formation of an adduct upon incubation of Flag-SENP5 with HA-SU1-VS, indicating that it had little reactivity toward this paralogue. These findings are completely consistent with earlier observations that mammalian SENP5 has a strong preference for SUMO-2/3 [15], [16], [17], and indicate that this preference is conserved amongst vertebrates.

### Developmental Patterns of *Xenopus laevis* SENPs

**Figure 2:**
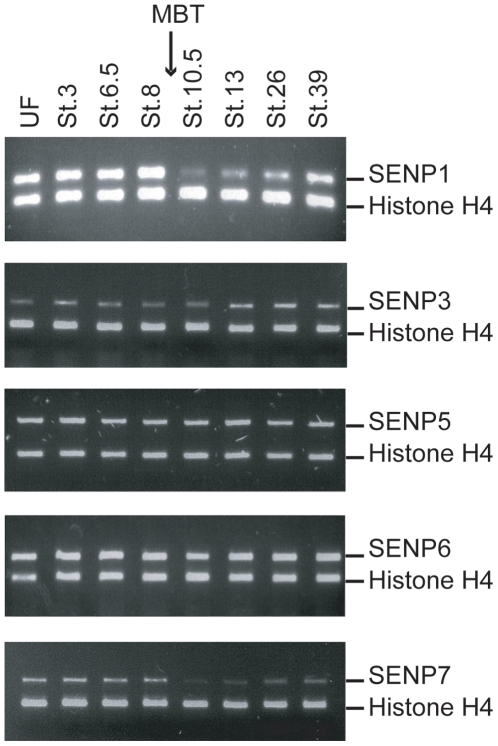
RT-PCR assay showed that mRNA levels for different SENPs family proteins vary during developmental stages. Total RNA from whole animals at the indicated stages was analyzed by RT-PCR using specific primers for different SENPs and Histone H4 (control). The interval corresponding to the midblastula transition (MBT) is indicated by the arrow. UF indicates samples from unfertilized eggs, while individual stages are indicated numerically, proceeded by the abbreviation St.

We examined the profile of expression for each of the SENP proteins in staged whole *Xenopus laevis* embryos by RT-PCR (Figure 2). We observed two distinct patterns of mRNA abundance: Three of the SENPs (SENP3, 5, 6) were relatively constant in abundance relative to a control mRNA that is constitutively expressed during early frog development (Histone H4). By contrast, two Ulp/SENPs (SENP1, SENP7) showed dramatic changes in abundance at the midblastula transition (MBT), a period during *Xenopus* development at which the cell cycle is remodeled, many maternal mRNAs are degraded and zygotic transcription is initiated [42], [43]. Our findings regarding SENP1 mRNA abundance are largely consistent with an earlier report [30], which showed decreased levels of SENP1 mRNAs slightly before the MBT. The levels of other SENP mRNAs have not been reported. This finding indicates that mRNAs encoding individual *Xenopus* SENPs are differentially regulated during early development.

**Figure 3:**
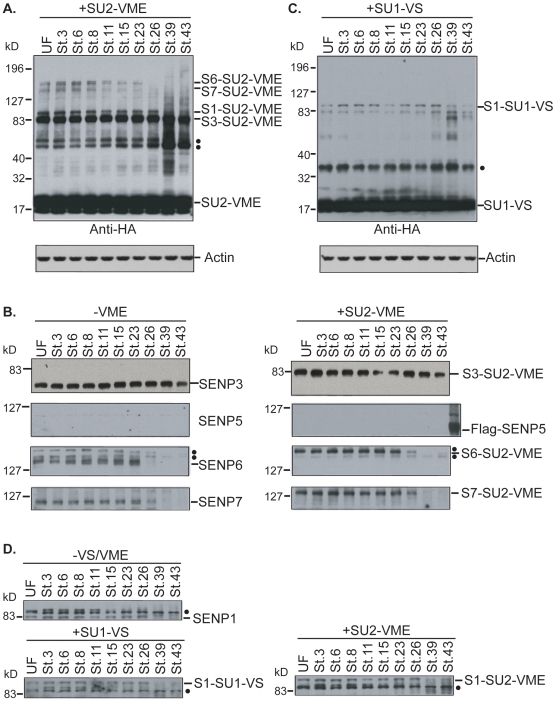
Developmental regulation of *Xenopus* SENP levels. For all panels, bands corresponding to unreacted SENPs are indicated with the full name of each protein. SENPs within adducts are indicated with the following abbreviations: S1 = SENP1. S3 = SENP3. S6 = SENP6. S7 = SENP7. SUMO moieties within adducts are indicated with the following abbreviations: SU1 = SUMO-1. SU2 = SUMO-2. UF indicates samples from unfertilized eggs, while individual stages are indicated numerically, proceeded by the abbreviation St. (**A**) Equal amount of protein from embryos of the indicated stages were incubated with excess HA-SU2-VME. Reactions were subjected to SDS-PAGE and Western blotting with anti-HA or -Actin antibodies. “▪” indicates unidentified bands. (**B**) Equal amount of protein from embryos of the indicated stages were incubated with excess HA-SU2-VME (right panels) or without HA-SU2-VME (left panels). The reactions were subjected to SDS-PAGE and Western blotting with anti-SENP3, SENP5, SENP6 and SENP7 antibodies. While we did not observe detectable bands on the blot with the anti-SENP5 antibodies, *in vitro* translated Flag-SENP5 was clearly recognized by this antibody (additional lane in right panel). (**C**) Equal amount of protein from embryos of the indicated stages were incubated with excess HA-SU1-VS. Reactions were subjected to SDS-PAGE and Western blotting with anti-HA or -Actin antibodies. “▪” Indicates unidentified bands. (**D**) Equal amount of protein from embryos of the indicated stages were incubated with excess HA-SU2-VME (right panel), HA-SU1-VS (lower left panel) or without addition (upper left panel). The reactions were subjected to SDS-PAGE and Western blotting with anti-SENP1 antibodies.

To determine whether SENP protein levels are likewise regulated, we examined changes in their levels both by Western blotting and by reactivity with vinyl methyl ester derivatives of tagged SUMO-1 and -2 (HA-SU1-VME and HA-SU2-VME; [44]). In all cases, actin was used as a loading control to assure that equivalent amounts of protein were loaded in each lane. In a manner similar to their vinyl sulfone counterparts, HA-SU1-VME and HA-SU2-VME react specifically and covalently with active-site cysteine thiol residues of Ulp/SENPs (Figure 3). As expected, the pattern of HA-SU2-VME reactivity found in unfertilized eggs was very similar to the pattern observed for HA-SU2-VS (Figure 3A). The band corresponding to SENP3 remained prominent through Stage 43 of development (prefeeding tadpole) [45]. Consistent with this finding, antibodies against *Xenopus* SENP3 recognized a protein on Western blots that persisted through Stage 43. This band could be confirmed to be a SUMO-2/3 specific protease because it clearly shifted upon incubation with HA-SU2-VME (Figure 3B) although not with HA-SU1-VME (data not shown).

SENP6 and SENP7 showed patterns similar to SENP3, although the levels of these proteins were initially lower, as judged by HA-SU2-VME reactivity (Figure 3). Interestingly, SENP6 and SENP7 decreased in abundance around Stage 26 (tail bud stage), with SENP6 levels dropping slightly before SENP7; both proteins were below detection by Stage 39. On the other hand, we did not see a substantial level of SENP5 expression during early *Xenopus* development. No band corresponding to SENP5 became apparent on blots of HA-SU2-VME reactive products using anti-HA antibodies (Figure 3A). Moreover, although polyclonal antibodies against *Xenopus* SENP5 could clearly recognize *in vitro* translated SENP5, Western blots did not show substantial levels at any stage (Figure 3B). Thus, much lower levels of SENP5 protein were found during development even though its mRNA was constitutively present.

The most prominent HA-SU1-VME reaction product corresponded to SENP1 (Figure 3C). It is possible that minor bands corresponded to SENP1 breakdown products or that they resulted from reaction with other SENPs. However, Western blots with antibodies against other SENPs showed no significant shift toward reduced mobility forms upon incubation with HA-SU1-VME (data not shown), indicating that the bulk of these proteins had not formed adducts with HA-SU1-VME. The SENP1-HA-SU1-VME adduct consistently showed a dip in abundance around Stage 11 (gastrula), but returned higher levels during Stage 15-26, in parallel with modulations of its mRNA. Consistent with this finding, antibodies against SENP1 recognized a band that showed adduct formation with HA-SU1-VME as well as HA-SU2-VME (Figure 3D), and that showed a comparable variation in abundance during development. SENP1 protein abundance declined after Stage 26.

These findings collectively indicate that individual *Xenopus* SENP proteins are differentially regulated during early development. Some of them (SENP1, SENP3 and SENP6) showed correspondence between the mRNA and protein levels through Stage 26. Changes in SENP1 mRNA and protein levels around the MBT were particularly striking, and may suggest that SENP1 undergoes turnover and re-synthesis around this time. While the SENP7 mRNA showed changes in abundance that were similar to the SENP1 mRNA, SENP7 protein levels did not change in close parallel. After Stage 26, SENP1, SENP6 and SENP7 protein levels all declined (Figure 3), despite the persistence of corresponding mRNAs. It is thus possible that these SENPs are subject to post-transcriptional regulation during later development. Taken together, our data show that individual SENPs were differentially regulated during embryogenesis, in patterns that could involve both transcriptional as well as posttranscriptional mechanisms.

### Developmental Requirement for SENP3

**Figure 4:**
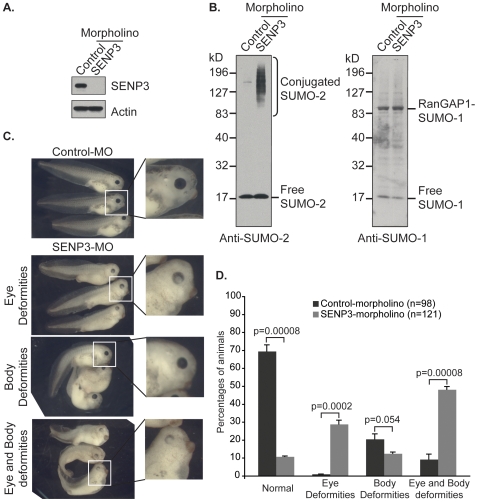
SENP3 is essential for development of *Xenopus laevis* embryos. (**A**) One cell embryos were injected with morpholinos, and subsequently harvested at Stage 39. Equal amounts of protein from Control-MO (left) or SENP3-MO (right) embryos were subjected to SDS-PAGE and Western blotting with anti-SENP3 or -actin antibodies. (**B**) Samples as in (**A**) were subjected to SDS-PAGE and Western blotting with anti-SUMO-2 and –SUMO-1 antibodies, as indicated. Note the substantial accumulation of high molecular weight SUMO-2-conjugated species after SENP3 knockdown, while there is essentially no change in SUMO-1 conjugates. (**C**) Morphological defects of SENP3-MO injected embryos were categorized according to defects in body patterning and eye development, as indicated (lower three panels). The morphology of embryos injected with Control-MO is shown in the upper panel. (**D**) Quantitation of morphological body deformities of SENP3-MO injected embryos. Bars indicate standard error of the mean values. *p* values are indicated.

Given that SENP3 was the most abundant SUMO protease in early embryos, we wished to determine how SENP3 expression might contribute to development. To address this question, we utilized morpholinos to knockdown expression of SENP3 protein. Morpholinos are synthetic oligonucleotides that have enhanced stability by virtue of the fact that they have morpholines rather than ribose rings within their backbone [46]. Embryos injected with control morpholinos (Control-MO) showed normal levels of SENP3 at Stage 39, as assessed by Western blotting with anti-SENP3 antibodies, while embryos injected with morpholinos directed against SENP3 mRNA (SENP3-MO) showed a dramatic decrease in SENP3 levels (Figure 4A). Western blotting of these embryos with antibodies against SUMO-2/3 showed a corresponding increase in the levels of high molecular weight conjugated species, while antibodies against SUMO-1 did not show detectably altered patterns, indicating that the loss of SENP3 causes a substantial shift in the abundance of SUMO-2/3 but not SUMO-1 conjugates (Figure 4B). This observation is consistent with the *in vitro* paralogue preference of SENP3 (Figure 1; Table 1).

We assessed whether this loss of SENP3 caused any change in early embryonic development. At Stage 39, around 90% SENP3-MO-injected embryos showed deformities in body structures, ocular structures or both (Figure 4C). In comparison, less than 30% of Control-MO-injected embryos showed similar deformities. Considering these features separately: 31% of control embryos showed defects in body structure. The level of body defects was enhanced roughly two-fold in the SENP3-MO-injected embryos, to around 61% (Figure 4D). A more dramatic difference was observed in eye development; only 9% of the Control-MO-injected embryos showed ocular defects. By contrast, 77% of the SENP3-MO-injected embryos showed ocular defects, roughly a 8.5-fold increase over the control group (Figure 4D). We were not able to rescue the developmental defects of SENP3-MO-injected embryos through microinjection of mRNA encoding SENP3. Interpretation of our results may therefore be limited by some possibility that the SENP3-MO also disrupts the expression of other mRNAs. More likely, however, this failure to rescue may reflect a requirement for high levels of SENP3 protein expression or for precise regulation of SENP3 expression that could not be recapitulated by the exogenous mRNA. Despite these limitations, our findings collectively suggest that SENP3 is essential for the maintenance of an appropriate spectrum of SUMOylated species during early development, and they are consistent with the notion that disruption of this spectrum has severe consequences for developing embryos, particularly for ocular structures.

### Disruption of Gene Regulation in SENP3-Depleted Embryos

**Figure 5:**
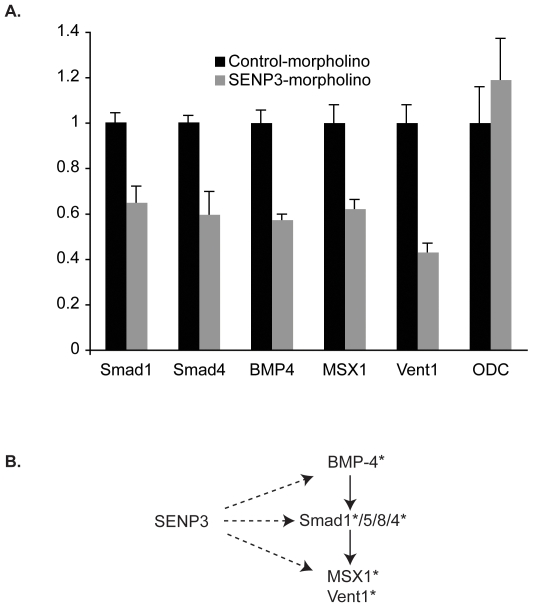
Inhibition of TGF-Beta signalling by SENP3 knockdown. (**A**) One cell embryos were injected with morpholinos, and harvested at Stage 39. The cDNA was generated from total RNA of tadpoles. The levels of expression for the indicated mRNAs were evaluated for Control-MO or SENP3-MO populations by qRT-PCR, and normalized to that of the control gene, elongation factor-1α, and the mean of the Control-MO was set to 1. One non-regulated gene, ornithine decarboxylase (ODC), was analyzed by qRT-PCR to confirm equivalent inputs of total mRNA. For individual genes, three independent samples were analyzed, and all comparisons had *p* values of <0.05. (**B**) Schematic representation of BMP signalling. SENP3 depletion disrupts expression of BMP target genes, MSX1 and Vent1. Asterisks indicate genes that are downregulated in the absence of SENP3.

Given the links between SUMOylation and the TGF-β pathway [32], [33], [34], [35], [36], [37], [38], we speculated that the defects we observed in eye development might reflect disregulation of this signaling cascade. We used quantitative real time PCR (qRT-PCR) of RNA from Stage 39 embryos to examine changes in expression of TGF-β pathway components and target genes in response to SENP3 depletion. We observed downregulation of signaling components within pathways controlled by the Bone Morphogenic Proteins (BMPs) (Figure 5): BMPs are the largest group of proteins within the TGF-β superfamily, and they have been particularly implicated in eye development [47]. We examined the expression of BMP4, which is expressed in forming *Xenopus* retina [48], and observed that its abundance and mRNAs encoding Smad signaling components (Smad1, Smad4) associated with the BMP pathway were sensitive to SENP3 depletion. Consistent with a downregulation of BMP4-mediated signaling, expression of the BMP4 targets Msx1 [49] and Vent1 [50] were decreased. Comparable levels of a control mRNA (ornithine decarboxylase (ODC)) in Control-MO- and SENP3-MO-injected embryos indicated that the effect of SENP3 depletion on the BMP4, Smad1, Smad4, MSX1 and Vent1 mRNAs was not due to a general loss of mRNA transcription or global mRNA destabilization,

Our findings support a role for SENP3 in enhancing the expression of BMP4 pathway targets, acting at one or multiple steps in the BMP4 pathway (Figure 5B). While these findings may help to account for the developmental phenotypes of SENP3-depleted embryos, they do not provide a direct mechanism of action for SENP3 in this pathway. It remains possible that the action of SENP3 might be relatively indirect, as other aspects of gene expression and particularly ribosome biogenesis are likely to be altered under these circumstances [17].

### Summary

Our observations provide a profile of Ulp/SENP expression and specificity through early vertebrate development in *Xenopus laevis*, showing that these enzymes are differentially regulated at the mRNA and protein levels (Figure 2, 3). We have further shown that the most abundant Ulp/SENP, SENP3, is particularly important for eye development, possibly through regulation of BMP signaling (Figure 4, 5). Cumulatively, these observations suggest that the SUMO pathway is developmentally regulated, and that the activity of individual Ulp/SENPs may play relatively specific roles during embryogenesis.

## Materials and Methods

### Chemicals and Reagents

Horseradish peroxidase (HRP) conjugated secondary antibodies (donkey anti-rabbit IgG and sheep anti-mouse), and NHS-Sepharose and Protein-A Sepharose were from Amersham Biosciences (Piscataway, NJ). Goat anti-rat IgG HRP conjugated secondary antibody was from Pierce Chemical (Rockford, IL), goat anti-Rat monoclonal anti-HA (3F10) was from Roche Diagnostic Corporation (Indianapolis, IN), mouse anti-flag M2 and anti-Actin antibodies were from Sigma-Aldrich Corporation (St. Louis, MO). All other reagents were obtained from Sigma-Aldrich Corporation (St. Louis, MO) unless otherwise stated.

### SENP cDNAs and Reverse Transcription-Polymerase Chain Reaction (RT-PCR)

Accession numbers for *Xenopus laevis* cDNAs corresponding to Ulp/SENPs were: SENP1: AF526893; SENP3: FJ416373; SENP5: EU275408; SENP6: FJ416371; SENP7: FJ416372.

For analysis of mRNA levels (Figure 2), RT-PCR was performed as described [51]. Total RNA was prepared at the indicated stages using TRIZOL reagent (Invitrogen, Carlsbad, CA). RT-PCR was performed according to the supplier's instructions with 200 ng of total RNA as the template, by using Super-Script One-Step RT-PCR with Platinum Taq (Invitrogen). RT-PCR products were run on 2% agarose gels, visualized with ethidium bromide staining under UV lights, and photographed with Kodak imaging system (Gel Logic 100 Imaging System, Kodak, New Haven, CT).

Primers were as follows: SENP1 ^5′^GATTTCATAGTTTCTCTAGTCGTATTT^3′^ and ^5′^CAGGAAACTGAAAGTTGAAGGC^3′^ [30]. SENP3 ^5′^CTGCAGACGCTCGAAATAGAGAAG^3′^ and ^5′^AGCGCTTGTACAGACCCCAGTATAG^3′^ . SENP5 ^5′^AAAGCAGATGATTGGTCAGAAGATG^3′^ and ^5′^AATGTTTTTCAAGGAAATGGGTTTT^3′^ . SENP6 ^5′^TTGGATCGATCTCAGTCAAAGAAAG^3′^ and ^5′^TTTCTCCAAGCTTTTTACTGGTTCC^3′^ . SENP7 ^5′^-AAAGCAGATGATTGGTCAGAAGATG^3′^ ^5′^AAGTCATACGGCACTTCTTCACTTG^3′^ . Histone H4 (loading control) ^5′^CGGGATAACATTCAGGGTATCACT^3′^ and ^5′^ATCCATGGCGGTAACTGTCTTCCT^3′^ [51].

### Antibodies and Immunological Methods

Rabbit anti-*Xenopus* SENP3 antibodies were as described [17]. A cDNA encoding a fragment of *Xenopus* SENP1 (amino acid 1–418) was subcloned between the *BamHI* and *SalI* restriction sites of pET30a. A cDNA encoding a fragment of *Xenopus* SENP5 (amino acid 327–500) was subcloned between the *BamHI* and *SalI* restriction sites of pGEX-4T1. A cDNA encoding a fragment of *Xenopus* SENP6 (amino acid 1–300) was subcloned between *BamHI* and *SalI* restriction sites of pET28a, and a cDNA encoding a fragment of *Xenopus* SENP7 (amino acid 1–200) was subcloned between the *BamHI* and *SalI* restriction sites of pET28a. Recombinant proteins were expressed from each of these constructs in *E. coli* [BL21 (DE3)] and purified over glutathione Sepharose 4 Fast Flow beads from GE Healthcare (Piscataway, NJ) or Ni-NTA agarose beads from Qiagen (Valencia, CA) according to manufactures' instructions. The recombinant proteins were injected into rabbits for the production of polycolonal antibodies (Pacific Immunology, Ramona, CA). In all cases, antisera were purified using antigens coupled to NHS-activated Sepharose 4 Fast Flow beads (GE Healthcare) as described previously [52].

For immunoblotting, anti-mouse IgG–HRP was used for FLAG- and HA-epitope and actin visualization. Anti-rabbit HRP was used for visualization of all other antibodies. Primary antibodies were used at a dilution of 1∶1000, and secondary antibodies were used at a dilution of 1∶5000 in PBS buffer with 0.1% tween 20 and 5% skim milk.

### SENP3 Morpholino Microinjection and qRT-PCR

The MO against SENP3 (CCGGATAACAGCCGTAGTGTCTCCC) and a standard control MO (CCTCTTACCTCAGTTACAATTTATA) were purchased from Gene Tools LLC. Morpholino oligos were suspended in sterile water. Fertilized *Xenopus* eggs were dejellied in 0.1x Marc's modified ringer solution (0.1x MMR: 0.5 mM HEPES, pH 7.6, 10 mM NaCl, 0.2 mM KCl, 0.1 mM MgCl_2_, and 0.2 mM CaCl_2_) containing 2% L-cysteine, washed extensively with 0.1x MMR and kept in 0.1x MMR plus 6% Ficoll 400. One-cell embryos were injected with 9.2 nl (90 ng) of Control-MO or SENP3-MO using a Nanoinject II (Drummond Scientific Company). Dead or abnormal embryos were removed after 4 hours. The embryos were kept at 18°C overnight and transferred to 0.1x MMR at room temperature thereafter. Phenotypes were analyzed at day 3. Proteins were extracted from whole embryos as described below and analyzed by Western blotting, using actin as a loading control (Figure 4).

For analysis of gene expression, total RNA from three independent samples was extracted from whole *Xenopus* embryos, cDNA was prepared, and real-time quantitative RT-PCR (qRT-PCR) was performed as described [53]. Mixed total RNA isolated from tadpoles of all stages spanning the entire metamorphosing period was reverse transcribed to serve as standard samples to produce a standard curve for the quantitative PCR analysis, where the cDNA was diluted at series of 1∶1, 1∶3, 1∶9, 1: 27, 1∶81, and 1∶243. All of the quantitative PCRs were carried out with a quantitative PCR machine (Model 7000 Sequence Detection System; Applied Biosystems). A set of primer specific for elongation factor-1α (EF-1α) was used as a control for RNA input of each sample, and the expression level of the gene of interest within each sample was normalized to that of EF-1α. Primers were as follows: Smad1 ^5′^GTCTTGCCACCTGTCCTTGTTCCAC^3′^ and ^5′^GGCATATGTGGCTCGCTTGGCT^3′^ . Smad4 ^5′^CAGACCTTCACAAGAATGAACTGAAGC^3′^ and ^5′^TCTATTCCAGGGGATACAACTCGTTCG^3′^ . Vent1 ^5′^AGGAGGCAACAGATGGAAAGGAC^3′^ and ^5′^TGGCATATTTGGTTGGGGGCAGTG^3′^ . Msx1 ^5′^ACTGGTGTGAAGCCGTCCCT^3′^ and ^5′^GGTCCCGGTCTCTCCCAGGT^3′^ . BMP4 ^5′^TGGAGATTGTCCATTTCCCTTGGC^3′^ . ^5′^GGGGACGCAGCATGCTTTTGG^3′^ . ODC ^5′^GCCATTGTGAAGACTCTCTCCATT^3′^ , ^5′^ATCCGCTCGGGGGAAACTCC^3′^ , and EF-1α: ^5′^CTATCCACCGCCAAACATCT^3′^ and ^5′^CCATCTCAGCAGCTTCCTTC^3′^ .

### Analysis of SENP5 Paralogue Specificity

The plasmid for expression of Flag-tagged *Xenopus* SENP5 (Flag-SENP5) was as described [17]. An inactive SENP5 lacking a catalytic cysteine residue (Flag-SENP5-C/S) was prepared by site mutation PCR using PfuUltra High-Fidelity DNA Polymerase (La Jolla, CA) with primers ^5′^CAGAAGAATGACAGTGACTCCGGTGTGTTTGTGCTTC^3′^ and ^5′^GAAGCACAAACACACCGGAGTCACTGTCATTCTTCTG^3′^ .

Immunoprecipitation was performed as described [17]. Briefly, FLAG-SENP5 and Flag-SENP5-C/S mRNAs were transcribed using an mMessage mMACHINE T7 kit (Ambion). The mRNAs were translated in *Xenopus* Egg Extracts (XEEs) [54]. 25 µl of the reaction was then mixed with 25 µl interphase XEE. The mixture was diluted five-fold with buffer A (250 mM sucrose, 2.5 mM MgCl_2_, 1 mM DTT, 50 mM KCl, 100 mM HEPES pH 7.7), and clarified by centrifugation at 15,000 g for 15 min. Mouse anti-flag antibody (M2, Sigma) or non-specific mouse IgG was added to aliquots of the clarified supernatant, and incubated for 1 hour on ice before addition of 15 µl pre-blocked Protein G Sepharose (GE Healthcare). After 1 hour at 4°C with rotation, the beads were pelleted, washed with buffer A, and eluted with 25 µl 0.1 M glycine, pH 2.3. The precipitated proteins were subjected to SDS-PAGE, transferred to the membrane and blotted with antibodies to Flag and HA, respectively.

### *Xenopus* Egg Extracts and Embryos

*Xenopus laevis* were purchased from Nasco Inc (Fort Atkinson, WI). All procedures were performed according to guidelines set by the NICHD animal use and care committee. Low-speed extracts of *Xenopus laevis* eggs were prepared as described [54]. Tadpoles were obtained by *in vitro* fertilization, and staged as described [45], [51]. For analysis of protein levels, 6 embryos at each stage were lysed by pipetting in 120 µl of buffer [20 mM HEPES, pH 7.5, 5 mM KCl, 1.5 mM MgCl_2_, 1 mM EGTA, 10 mM β-glycerophosphate, 50 mM NaCl, 0.1% NP-40, and protease inhibitors (5 mg/ml of each leupeptin, pepstatin and chymostatin, and 1 mM of AEBSF)]. After centrifugation at 15,000 g for 15 min at 4°C, the proteins level of supernatant was determined by Nanodrop (Thermo Scientific). Equal amounts of proteins were diluted with 5x SDS-loading buffer and then subjected to SDS-PAGE and immunoblotting, with actin as a loading control.

### HA-SUMO-VS or VME Experiments

VS derivatives of SUMO-1 and -2 were prepared and reactions were performed as described previously [22]. 5 ng/µl HA-SUMO-VS or VME was added to equal amounts of prepared lysates from embryos or XEEs, and allowed to react for 30 minutes at room temperature. The reactions were terminated by the addition of 5x SDS sample buffer and analyzed by SDS-PAGE and immunoblotting with the indicated antibodies. For mass spectrometry, proteins associated with HA-SU1-VS and HA-SU2-VS were purified using an anti-HA affinity matrix (Roche). Proteins were eluted with 0.1 M glycine-HCl (pH 2.5) neutralized with 1M Tris-HCl pH 8.0 containing 6 mM sodium deoxycholate and overnight precipitated with 10% trichloroacetic acid (TCA). TCA pellets were re-suspended in minimal volume of 0.2 N NaOH followed by sample buffer and subjected to SDS-PAGE, and the individual bands were cut out from gel and analyzed by mass spectrometry.

## Supporting Information

Figure S1 Identification of vinyl sulfone reaction products. A) The table indicates SENP proteins positively identified by mass spectrometric analysis in the first column, with GenBank acquisiton numbers (second column) and their predicted molecular mass (third column). For each protein, we indicate the number of predicted peptides found within the sample that match the theoretically calculated mass of tryptic peptides for the given protein (fourth column). We also indicate the number of number of predicted peptides found within the sample that match the theoretically calculated mass of tryptic peptides derived from HA-SU1-VS or HA-SU2-VS (fifth column). Note that in some cases we did not find peptides derived from SUMO proteins, although the corresponding bands were clearly recognized by anti-HA antibodies. B) Peptides from the major HA-SU1-VS adduct that match sequences in SUMO-1 and SENP1 are shown in red. C) Peptides from the major HA-SU2-VS adducts that match sequences in SUMO-2, SENP1, SENP3 and SENP6 and are shown in red. Note that SENP7 was not sequenced in these experiments. It was identified using specific polyclonal antibodies that recognized a HA-SU2-VS reaction product (see Figure 1, 3).
(0.03 MB DOC)

Figure S2 Rabbit SENP5 antibody recognizes *in vitro* translated Flag-SENP5. *In vitro* translated Flag-SENP5 was subjected to immunoprecipitation from XEEs using antibodies against the Flag or non-specific IgG. The purified proteins were subjected to SDS-PAGE and Western blotting using anti-Flag antibodies (left panel) or affinity purified rabbit anti-SENP5 antibodies (right panel).
(0.98 MB TIF)
