# Supplementary material for: Identification and Developmental Expression of Xenopus laevis SUMO Proteases
Source: PLoS One. 2009 Dec 24;4(12):e8462. doi: 10.1371/journal.pone.0008462 (PMC2794540; doi:10.1371/journal.pone.0008462)
Supplement: Figure S1 — Identification of vinyl sulfone reaction products. A) The table indicates SENP proteins positively identified by mass spectrometric analysis in the first column, with GenBank acquisiton numbers (second column) and their predicted molecular mass (third column). For each protein, we indicate the number of predicted peptides found within the sample that match the theoretically calculated mass of tryptic peptides for the given protein (fourth column). We also indicate the number of number of predicted peptides found within the sample that match the theoretically calculated mass of tryptic peptides derived from HA-SU1-VS or HA-SU2-VS (fifth column). Note that in some cases we did not find peptides derived from SUMO proteins, although the corresponding bands were clearly recognized by anti-HA antibodies. B) Peptides from the major HA-SU1-VS adduct that match sequences in SUMO-1 and SENP1 are shown in red. C) Peptides from the major HA-SU2-VS adducts that match sequences in SUMO-2, SENP1, SENP3 and SENP6 and are shown in red. Note that SENP7 was not sequenced in these experiments. It was identified using specific polyclonal antibodies that recognized a HA-SU2-VS reaction product (see Figure 1, 3). (0.03 MB DOC) [file pone.0008462.s001.doc]

**Figure S1.** **Identification of vinyl sulfone reaction products.**

A.

| **Protein** | **Accession** | **Mol.mass (Da)** | **SENP Peptides** | **SUMO peptides** | **HA-SU-VS adduct** |
| --- | --- | --- | --- | --- | --- |
| SENP1 | AF526893 | 70176.91 | 17 | 0 | SUMO-2 |
| SENP3 | FJ416373 | 52911.41 | 34 | 15 | SUMO-2 |
| SENP6 | FJ416371 | 123734.86 | 14 | 0 | SUMO-2 |
| SENP1 | AF526893 | 70176.91 | 17 | 3 | SUMO-1 |

**B. *HA-SU1-VS reaction product***

**SENP1**

MDDDVKEMVWESENNSTFKAHYKETAHNSSYAFNFQFPALQYKHFEISGMNSIHKPNFVSEKYERAIPPGDKMIPQQSSELTKGKQNGNGYAVLPAKGVSSQKPRVPRSAHMEARKLSGALNAASGKPNHTLASAYEKSFPFKNISCSASLIGPCRRSPKKTQRRFVSTVEETVREEEKEIYRQLLQVVTGKTFLSTKSTSILPPQVSRCLSSDNSISGQPVASSLSSLEPSSLDTESSCRTSFSYLQPSGQISEAILSNSTNFKVISDTQGASNQQLPKEKHAPSSQQSQGLDSPIVLDSPVVKPREPASQPFFHAELWIKELTSLFDSRSRERRRQIEEQEALALQLQKQRLQECSVQDSIDLHLRVPLEKEIPVTLIPKQEESPKPEEIEFPEITEVMEREIKRALFGGSQDQSLSEGYRLTITRKDIMTLHSLNWLNDEIINFYMNLLMERSKRKGLPTVHAFNTFFFTKLKSAGYQAVKRWTKKVDIFSMNILLVPIHLGVHWCLAVVDLRKKSITYFDSMGGLNNDACRILLQYLKQESVDKKGACFDSNGWTLTCKTSEEIPQQMNGSDCGMFACKYADYITKDKSITFTQHHMPYFRKRMVWEILHQKLL

**SUMO-1**

MSDQEAKPSSEDLGDKKDGGDYIKLKVIGQDSSEIHFKVKMTTHLKKLKESYRQRQGVPMNSLRFLFEGQRISDHQTPKELGMEEEDVIEVYQEQTGGHSTF

**C. *HA-SU2-VS reaction products***

**Band 1: SENP6**

MAGGGGSQLLEALDRSQSRKDGGFSNCSFYNSEDSEDDTEKDEANLLSLDESDDADLQITEQKPKHLRSGTARALGDSIKTYERRGRTLHFTALKGNAIGLNMLGASKKLGENAQNIPVTSGTIVQGRIFHHTNIPRSTVKTAAQRKEYPAHVQKVDSDQGRLHTSLKSENVQERKEDSNFESEPEKIKRKAQQRRHSSVPECDMSLTRTPQMCLTLPEGVRQAEELQFCTVCVKENKKTKCQSTESSQRLCKQPIALNELSPLPRPAIHQIDGRTAKLAFNVKSFYSSTCNQLIPNDFVQQYPANGKLASEGNTSKAAKNMRLRSAGSSEPNDPIVLSSDDEDNASTGSTNRIESISPRPADSACSSPAPSSGKVEAALKENSFTFEHKFCNSAIDAASTIALPRKAKMKDQFGNTVSRTPVKRRKVFAPETPPEPAPDANSNCESLVMNCRSVRIGSYYREAIEHVVFSMDFITIRWEVLPEDGNSQELVLYPSELTKCEWCAVRKLPVVFFQTLPAACQSLRSRLKLSRENGTGWYDCRATNLEEQFIVLIFENAPDMKATVGFETILYNIGIKNGIKNFFIKIPFEDANGRLVAFTKNSEDIPVMGSTQKENIEKNAATETRMKLRNSSQLQFFGDDEGDTHTVFVGPVEKLIVYPPPPAKGGISVTNEDLHCLNEGEFLNDVIIDFYLKYLVLEKLRKDADRIHIFSSFFYKRLNQRERRNLQPPANLTLQQRRHGRVKTWTRHVDIFQKDFIFVPLNEAAHWFLAVICFPGLEKPEHYPNPYYQGVSSTATVGEGDNTSASLPQNASETTLQGASSNSPLKKTLGKNTGTDAIDTLGPKRNHSLTRHRSRKSDQGLQEEDVSMKPVFSDSETSNNDINGATNECKTPVKPRDGLHRIQISYTETTDDTKKSEDEFIDFSDDQDNMDECSDDASLTDENYTSEAGKWHLKQFFCKQPCILLMDSLRGPSRSTVVKTLREYLEVEWEVRKGSKRSFSKDVMKGSSTRVPQQNNLSDCGVYILQYVESFFENPIQSFDLPMNLMDWFPQQRMKTKREEICNLILTLQGLQSKEKKGNKEPSTMQHAAQEKPEPCISSGSD

**Band 2: SENP1**

MDDDVKEMVWESENNSTFKAHYKETAHNSSYAFNFQFPALQYKHFEISGMNSIHKPNFVSEKYERAIPPGDKMIPQQSSELTKGKQNGNGYAVLPAKGVSSQKPRVPRSAHMEARKLSGALNAASGKPNHTLASAYEKSFPFKNISCSASLIGPCRRSPKKTQRRFVSTVEETVREEEKEIYRQLLQVVTGKTFLSTKSTSILPPQVSRCLSSDNSISGQPVASSLSSLEPSSLDTESSCRTSFSYLQPSGQISEAILSNSTNFKVISDTQGASNQQLPKEKHAPSSQQSQGLDSPIVLDSPVVKPREPASQPFFHAELWIKELTSLFDSRSRERRRQIEEQEALALQLQKQRLQECSVQDSIDLHLRVPLEKEIPVTLIPKQEESPKPEEIEFPEITEVMEREIKRALFGGSQDQSLSEGYRLTITRKDIMTLHSLNWLNDEIINFYMNLLMERSKRKGLPTVHAFNTFFFTKLKSAGYQAVKRWTKKVDIFSMNILLVPIHLGVHWCLAVVDLRKKSITYFDSMGGLNNDACRILLQYLKQESVDKKGACFDSNGWTLTCKTSEEIPQQMNGSDCGMFACKYADYITKDKSITFTQHHMPYFRKRMVWEILHQKLL

**Band 3: SENP3**

MRHTPVQRHLPPFSPHCLARGKNEKLFYTGGDEIWDEEEEEEEEEGLRRACTPPPRQQRRGRRQRLRNLRYLYWGLYKRSAMLPLQWKLWGGLGGRRRRLWRKVREGGGCSVGVELDKSHGRGAEEVASVANHVADGETDSPHGLPNGFSESDPILITNVCSIGNQETPAINGPHPQEELTPPPEKEAPPIVTLTQEHVTCVQSILDQFLQTYGSLIPLGTEEVLEKLEDVFQEPFTTPARRTVVQHVIQSYQRHPGNAMLRGFRVTYKRHVLSMDDLGTLYGQNWLNDQVMNMYGDLVMDAVPDKVHFFNSFFYDKLRTKGYEGVKRWTKNVDIFNKQLLLIPIHLEVHWSLVCVDVPNRTITYFDSQRTLNRRCPKHIAKYLQAEAVKKDRPEYVSGWTGLFKMNVARQNNDSDCGAFVLQYCKFLALGLPFTFGQQDMPKLRRQIYKELCHCKLAV

**SUMO-2**

MADDKPKEGVKTENNDHINLKVAGQDGSVVQFKIKRHTPLNKLMKAYCERQGLSMRQIRFRFDGQPINETDTPAQLEMEDEDTIDVFQQQTGGSF
