# Supplementary figures and images for: Identification and Developmental Expression of Xenopus laevis SUMO Proteases
Source: PLoS One. 2009 Dec 24;4(12):e8462. doi: 10.1371/journal.pone.0008462 (PMC2794540; doi:10.1371/journal.pone.0008462)

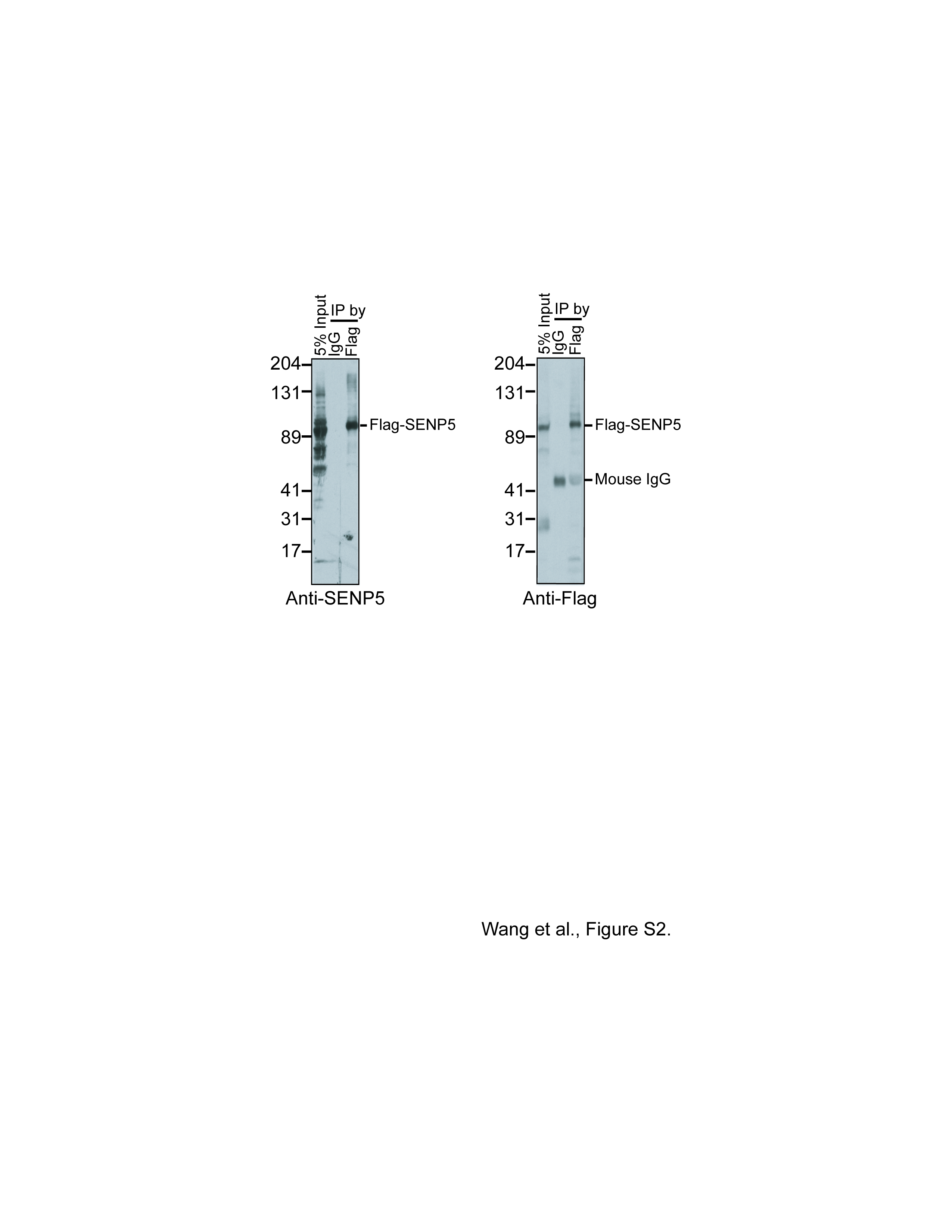

Supplement: Figure S2 — Rabbit SENP5 antibody recognizes in vitro translated Flag-SENP5. In vitro translated Flag-SENP5 was subjected to immunoprecipitation from XEEs using antibodies against the Flag or non-specific IgG. The purified proteins were subjected to SDS-PAGE and Western blotting using anti-Flag antibodies (left panel) or affinity purified rabbit anti-SENP5 antibodies (right panel). (0.98 MB TIF) [file pone.0008462.s002.tif]
